# *Fusobacterium nucleatum* and Its Impact on Colorectal Cancer Chemoresistance: A Meta-Analysis of In Vitro Co-Culture Infections

**DOI:** 10.3390/cancers17193247

**Published:** 2025-10-07

**Authors:** Katie R. Risoen, Claire A. Shaw, Jeremy Chien, Bart C. Weimer

**Affiliations:** 1 Population Health and Reproduction, 100K Pathogen Genome Project, School of Veterinary Medicine, University of California, Davis, CA 95616, USA; krrisoen@ucdavis.edu (K.R.R.); clashaw@ucdavis.edu (C.A.S.); 2 School of Medicine, University of California, Davis, Sacramento, CA 95817, USA; jrchien@ucdavis.edu

**Keywords:** *Fusobacterium nucleatum*, colorectal cancer, chemoresistance, meta-analysis, inflammation, metastasis

## Abstract

**Simple Summary:**

Bacteria from the mouth are increasingly found in the gut and linked to worse outcomes in colorectal cancer, one of the leading causes of cancer death worldwide. One such bacterium, *Fusobacterium nucleatum*, has been shown to interfere with cancer treatment, contributing to chemoresistance. This study brings together data from several independent experiments where colorectal cancer cells were grown with *Fusobacterium* to identify consistent patterns in how the bacteria influence cancer biology. The combined analysis revealed that the bacterium activates immune and antiviral pathways, alters how cells repair DNA, induces metastasis, and may reduce the effectiveness of common cancer drugs. These findings reinforce the role of bacteria in cancer progression and drug resistance, providing a valuable resource for future research into improving treatment outcomes.

**Abstract:**

Introduction: *Fusobacterium nucleatum*, a common oral microbe associated with periodontal disease, has emerged as a significant prognostic indicator in colorectal cancer (CRC). This organism is notably enriched in CRC tissues and is associated with reduced survival times and relapse. Fusobacterium is implicated in encouraging the development of chemoresistance through diverse tumor-promoting pathways that are increasingly being elucidated across molecular domains. Methods: This work uses a combined analysis of public data examining the role of *F. nucleatum* in CRC by investigating multiple transcriptomic datasets derived from co-culture infections in vitro. Results: In tandem with previously identified mechanisms known to be influenced by *F. nucleatum*, this analysis revealed that the bacterium activates multiple chemoresistance-associated pathways, including those driving inflammation, immune evasion, DNA damage, and metastasis. Notably, this study uncovered a novel induction of type I and type II interferon signaling, suggesting activation of a pseudo-antiviral state. Furthermore, pathway analysis (IPA) predicted altered regulation of several therapeutic agents, suggesting that *F. nucleatum* may compromise drug efficacy through transcriptional reprogramming. Conclusions: These findings reinforce the role of *F. nucleatum* in modulating host cellular pathways and support the hypothesis that bacterial association potentiates chemoresistance.

## 1. Introduction

Globally, colorectal cancer (CRC) is the fourth most diagnosed cancer and the second most frequent cause of cancer-related death [1], with a relative 5-year survival rate of 17% for distant metastatic stages [2]. This high mortality is attributed to the incidence of metastasis and chemoresistance in CRC [3]. The gut microbiome plays a critical role in human health, particularly in immune regulation and maintaining intestinal homeostasis [4]. Disruptions to the gut microbial community are associated with reduced effectiveness of chemotherapy and poorer clinical outcomes [5,6]. In the context of CRC, tumor tissues frequently exhibit microbiome dysbiosis, harboring distinct bacterial populations not found in adjacent healthy mucosa. Among these, *Fusobacterium nucleatum* often emerges as one of the most enriched taxa within colorectal tumors, drawing particular attention for its potential role in cancer progression and resistance to therapy [7,8].

*F. nucleatum* is a common oral microbe that plays a central role in periodontal disease [9]. However, the clinical understanding of *F. nucleatum* has expanded due to its isolation from the microbiome of tissues distant from the oral environment. Notably, this organism has been associated with cancerous tissues, suggesting that this microbe may participate in multiple negative outcomes in cancer development and treatment [10]. *F. nucleatum*’s influence on cancer has most extensively been studied in CRC, where it is becoming one of the most important microbe-related risk factors for CRC prognosis [11]. *F. nucleatum* has been identified to be significantly enriched in CRC carcinoma tissues compared to healthy adjacent tissues [7]. This pathogen is known to increase tumor proliferation and invasive activities of CRC cells, encouraging inflammation, immune evasion, DNA damage, and metastasis [11,12,13,14]. *F. nucleatum* participates in these activities by activating TLR4 signaling [15,16,17] and subsequently inducing oncogenic pathways, including Wnt/β-catenin [18,19], PI3K/Akt [20], and NF-κB [21,22]. Activation of these pathways stimulates robust pro-inflammatory cytokine production, including IL-1β, IL-6, IL-8, IL-17A, and TNF-α [12,13]. Moreover, *F. nucleatum* impairs tight cell junctions and promotes epithelial-to-mesenchymal transition (EMT) by binding and invading epithelial cells through FadA and Fap2 adhesins [23,24,25]. Additionally, *F. nucleatum* recruits myeloid-derived suppressor cells (MDSCs) and modulates tumor-associated immune populations by suppressing cytotoxic activity to foster an immunosuppressive tumor microenvironment [14,15,25,26].

Due to these various molecular modifications, *F. nucleatum* colonization has been linked to the development of chemoresistance in CRC [13]. *F. nucleatum* enrichment in the tumor-associated microbiome from CRC was found in patients with shorter survival times and increased relapses [27,28], suggesting that this pathogen may be a marker of poor prognosis. The use of antibiotic treatment to target anaerobic bacteria, including *F. nucleatum*, in CRC patients prior to surgical resection had a reduced risk of recurrence or death by 25.5% [29], further suggesting that prognosis is associated with the microbiome. *F. nucleatum* induces chemoresistance through multiple mechanisms, including activating autophagy, suppressing apoptosis, and regulating the expression of genes critical to drug response [13,28,30]. Through the establishment of a pro-inflammatory and immunosuppressive tumor microenvironment that promotes metastasis and facilitates DNA damage, *F. nucleatum* enhances the tumor’s susceptibility to the development of chemoresistance [13].

Some emerging studies have suggested *F. nucleatum* plays a role in engaging in viral reactivation and host antiviral defense pathways, a relatively underexplored mechanism with potential implications for chemoresistance [31,32]. For example, oral coinfections are prevalent in HIV-1 patients, where *F. nucleatum* has been found to induce latent HIV-1 reactivation through TLR2 and TLR9 [33]. Pathogen recognition receptors (PRRs), activated by pathogen-associated molecular patterns (PAMPs), induce the interferon system to initiate an antiviral response [34]. Specifically, *F. nucleatum* activates RIG-I, a key cytosolic PRR that prompts type I interferon production, through intracellular invasion and secretion of its nucleic acids [32,35]. Although these responses are classically antiviral, persistent activation within the tumor microenvironment can paradoxically contribute to immune dysregulation, inflammation, and resistance to apoptosis [36,37]. In cancer models, chronic activation of the interferon pathway has been linked to immune evasion and a poor response to chemotherapeutic agents [38,39,40].

The widespread alteration of the cell signaling within CRC tissues clouds the mechanistic understanding of how this organism participates in the multiple and complex signals to the cell. This gap in understanding, coupled with increasing recognition that tissues once thought to be sterile have a robust and active microbiome, opens many questions about therapeutic options. It is estimated that microbial pathogens promote tumorigenesis in 15–20% of cancer cases [41], yet the complete repertoire for how the microbiome influences cancer progression and resistance remains underdeveloped. This study conducted a meta-analysis of all publicly available studies, allowing a direct comparison of the impact of *F. nucleatum* in-vitro. This work aims to help identify multi-step complex networks of signals that are important yet largely overlooked for *F. nucleatum* interactions with CRC. This was done by examining multiple transcriptomic datasets from in-vitro co-culture of *F. nucleatum* with CRC cell lines to find the capacity of *F. nucleatum* to disrupt key regulatory cell functions to initiate a multitude of cascades that lead to chemoresistance as well as antiviral responses, which may also contribute to chemoresistance. These findings reflect a growing understanding that orally derived *F. nucleatum* may disseminate to distal tissues and potentiate or exacerbate severe diseases like CRC. More specifically, this study highlights multi-step complex pathways related to chemoresistance that may be regulated by interactions with *F. nucleatum.*

## 2. Materials and Methods

### 2.1. Search Strategy

A systematic search of the NCBI Gene Expression Omnibus (GEO) database [42] was conducted on 20 March 2025, to identify transcriptomic datasets examining the impact of *F. nucleatum* in cancer-related contexts. The search terms used were: (“cancer” OR “tumor” OR “carcinoma”) AND (“*Fusobacterium*” OR “Fusobacteria” OR “*F. nucleatum*”).

### 2.2. Selection Criteria

Search results were filtered using the Top Organism: *Homo sapiens* and Entry Type: Series. Duplicate entries were removed prior to analysis. Studies were subsequently manually reviewed and selected based on the following criteria: (1) gene expression profiling from RNA sequencing; (2) CRC cell lines as the model system; and (3) co-culture infection of CRC cell lines with *F. nucleatum*. Datasets that did not meet these conditions or lacked appropriate control samples were excluded.

### 2.3. Data Extraction and Synthesis

The ‘Series RNA-seq raw counts matrix’ file was downloaded from GEO for each dataset. The raw count matrices were combined into a single file, with sample labels assigned as either the control group or the *F. nucleatum* treatment group based on the original study design. This combined raw counts matrix containing all four datasets was then used for differential expression analysis and interpretation. DESeq2 [43] was used within R (version 4.4.1) to analyze the merged dataset. Counts were first normalized using count normalization within DESeq2, then the normalized count table was used to determine differentially expressed genes between *F. nucleatum*-treated host cells compared to no bacteria added controls. This resulted in a table of log_2_FC values and associated significance values for *F. nucleatum*-treated cells compared to non-infected controls, which was used as the input for downstream analyses.

Merging datasets using this strategy strengthens the ability to detect genes that were consistently and significantly altered across multiple conditions, increasing the biological relevance of the findings. This filtering approach reduced noise but may exclude moderately changing genes that may be biologically important. The dataset was not included as a covariate, since the condition was fully nested within the dataset, and modeling it would eliminate the treatment signal. We acknowledge that unmodeled dataset effects remain a limitation of this approach, although consistent patterns across datasets support the biological relevance of the results. Datasets that could not have RNAseq counts downloaded from the GEO database were excluded from downstream analysis. Public metadata from each study were used to identify control and treatment samples (i.e., which samples were treated with *F. nucleatum*).

### 2.4. Data Synthesis and Analysis

The combined dataset was imported into Ingenuity Pathway Analysis (IPA) (Qiagen, Redwood City, CA, USA) for enrichment analysis and signal transduction visualization. Gene identifiers were mapped, and a cutoff of |*p*-value| ≥ 0.1 was applied. Core analysis was performed within IPA (Qiagen) to identify enriched canonical pathways, upstream regulators, causal networks, and disease/function annotations relevant to *F. nucleatum* interactions in CRC.

### 2.5. Statistical Analysis

Statistical significance and pathway predictions were evaluated using IPA (Qiagen). Enrichment and differential expression analyses were assessed using −log(*p*-value), z-score, and log_2_FC, reflecting the statistical significance and magnitude of gene expression changes. In addition to enrichment analysis, IPA (Qiagen) was used to determine predicted upstream regulator activity and pathway dynamics, computed through activation z-scores and −log(*p*-value), which quantify the direction and strength of predicted activation or inhibition based on concordance between observed and expected gene expression patterns. Hierarchical clustering and its accompanying dendrograms were created using MetaboAnalyst 6.0. Figures were made and designed in IPA, Adobe Illustrator (version 29.5; San Jose, CA, USA), and bioRender (bioRender, Toronto, ON, Canada).

## 3. Results

### 3.1. F. nucleatum Infection Outcome Depends on Experimental Parameters

**Figure 1:**
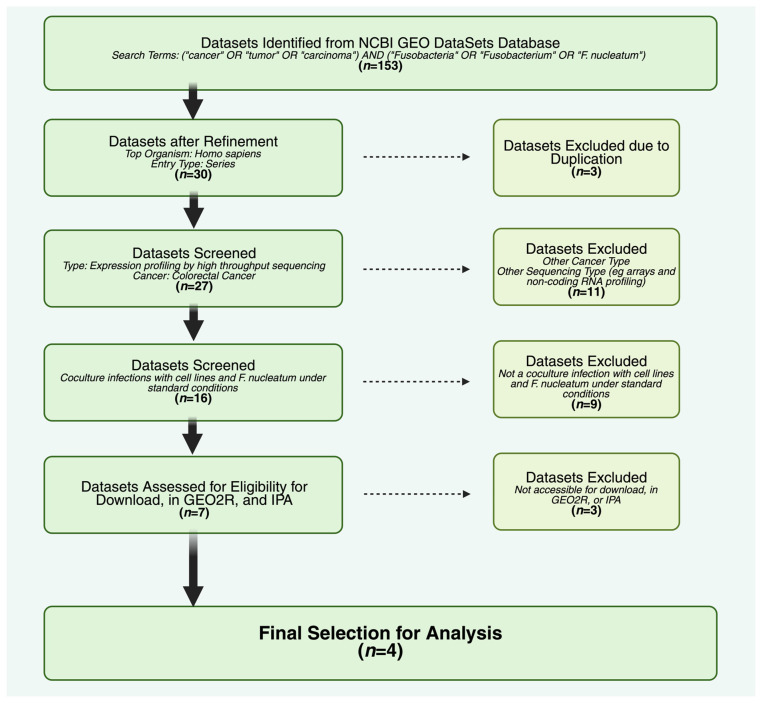
Dataset selection pipeline. After searching the NCBI GEO DataSets Database, filtering with our selected criteria, and removal of duplicates or inaccessible datasets, a final selection of four datasets continued with further analysis: GSE2454617, GSE173549, GSE90944, and GSE175593. *n* indicates the number of datasets.

**Figure A1:**
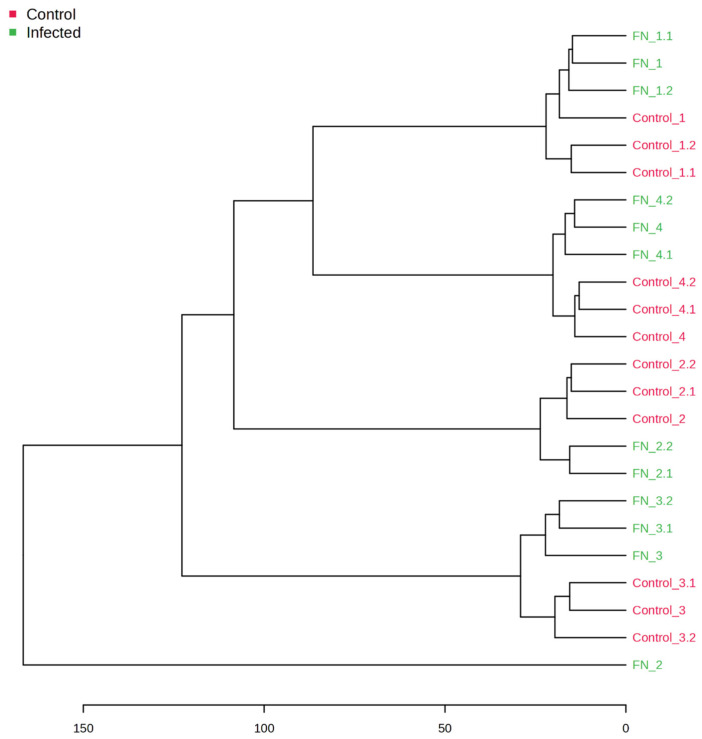
Dendrogram of datasets to indicate hierarchical clustering. FN indicates CRC cell lines cocultured with *F. nucleatum*, while Control indicates CRC cell lines that were not infected. The numbers following their identified experimental condition indicate the dataset, being 1–4, followed by the identification of the biological replicate in that experiment. Replicates grouped together based on their dataset, rather than experimental condition.

After screening all public datasets, four eligible datasets were identified that met the quality control settings used in this study and were available on NCBI GEO (Figure 1). Each dataset had its own cell line, infection time, multiplicity of infection (MOI), and overall concluding results, as reported in its associated paper (Appendix A, Table A1), establishing initial variability among the datasets. As these datasets contained only human RNA sequences, we could not directly confirm the presence or abundance of *F. nucleatum*. However, because each study was conducted in-vitro under controlled conditions with a defined MOI, we relied on the authors’ experimental design and documentation to assert bacterial exposure as defined in the publication. Hierarchical clustering was performed with the expectation that control and infection groups from these individual studies would cluster. This analysis revealed that datasets grouped together rather than treatment with *F. nucleatum*, indicating experimental design influenced the ultimate study outcome (Appendix A, Figure A1). Therefore, findings that were statistically significant represent those genes and pathways that were above the variation introduced by differences in the study design.

### 3.2. F. nucleatum Promotes Chemoresistance in Colorectal Cancer

**Figure 2:**
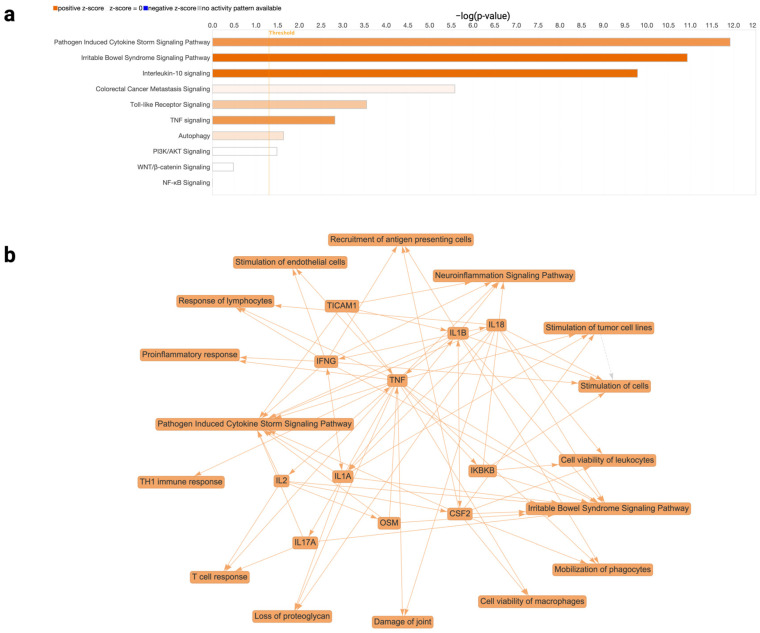
(**a**) Pathways related to cancer processes. There were enrichments in cancer-related pathways such as pathogen-induced cytokine storm signaling, irritable bowel syndrome signaling, interleukin-10 signaling, colorectal cancer metastasis signaling, toll-like receptor signaling, TNF signaling, and autophagy. However, oncogenic pathways PI3K/AKT signaling, WNT/β-catenin signaling, and NK-κB signaling were insignificant or inconclusive; (**b**) Graphical summary of the combined dataset. This summary indicates the most significant pathways, the most activated or inhibited pathways, key molecules and interactions, and predicted biological effects, providing a high-level overview of the dataset. Various cytokines have been identified, along with the biological processes involved in inflammation, immune dysregulation, proliferation, and tissue damage.

A combined dataset consisting of the four selected studies was run through the core analysis pipeline in IPA, provided in Supplemental File S1. Within this combined dataset, cancer-related inflammatory pathways were the most significantly activated pathways in response to *F. nucleatum* treatment (Figure 2a). Localized digestive tract-related pathways like those related to digestive system cancers (−log(*p*-value) = 19.247, z-score = +1.40) and to irritable bowel syndrome (−log(*p*-value) = 10.934, z-score = +3.528) were significantly activated with *F. nucleatum* addition. Further supporting this observation of broad inflammatory activation was the more specific induction of cytokine genes such as *CSF2*, *IL2*, *IL1A*, *IL1B*, *IL17A*, *IL18*, *IFNG*, and *TNF* (Figure 2b). Together, these observations indicate that *F. nucleatum* addition modulated the host infectious disease response, immune response, activation of inflammatory signaling, and stimulation of tumor proliferation, all of which may affect cancer onset, progression, and prognosis.

While the activation of inflammatory pathways from *F. nucleatum* association appeared as a consistent trend, the regulation of multiple cell death and migration pathways by *F. nucleatum* was inconsistent. Despite the significant activation of pathways involved in apoptosis and organismal death (−log(*p*-value) = 13.947, z-score = +1.807), those for survival (−log(*p*-value) = 10.055, z-score = +1.853) and migration of tumor cells (−log(*p*-value) = 6.777, z-score = +2.301) were also induced. Additionally, the oncogenic pathways Wnt/β-catenin signaling (−log(*p*-value) = 0.482, z-score = NaN) and PI3K/AKT signaling (−log(*p*-value) = 1.484, z-score = 0) were expected to be upregulated based on previous work [18,19,20]. However, this analysis found that *F. nucleatum* did not significantly affect their regulation at the pathway level. These seemingly contradictory findings suggest a complex interplay between cell death and tumor-promoting pathways that was not captured in the models used. Such complexity presents challenges when attempting to define this host–pathogen relationship and perhaps in part explains the confusion and contradictory evidence from in vivo studies [44].

#### 3.2.1. *F. nucleatum* Induces Chronic Inflammation

**Figure 3:**
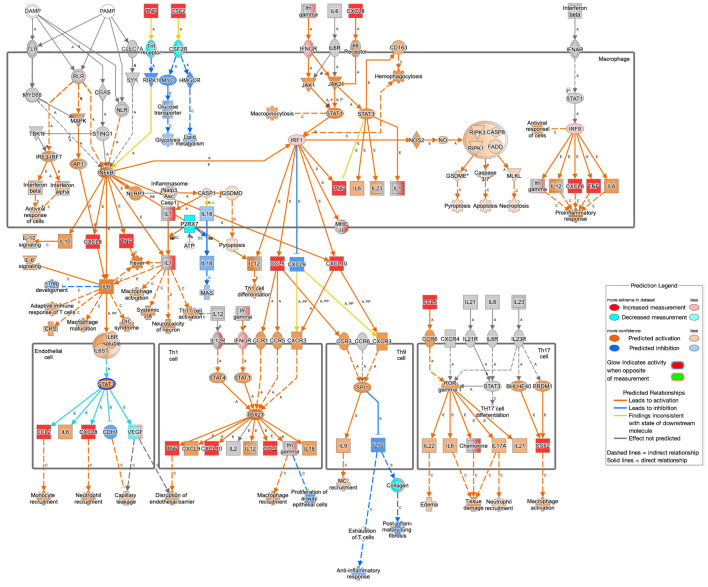
Pathogen-induced cytokine storm signaling pathway. Innate and adaptive immune pathways activated by pathogen-associated molecular patterns (PAMPs), cytokines, and chemokines. Upstream signals such as TNF, IL-6, IRF1, IRF9, and NF-κB stimulate downstream cascades that control tissue integrity disruption, cytokine production, cell recruitment, apoptosis, macrophage activation, and pro-inflammatory responses. Specifically, cytokines and chemokines *TNF*, *IL1B*, *CCL2*, *CCL5*, *CCL20*, *CXCL8*, *CXCL1*, and *CXCL10* were differentially expressed. * next to a gene name indicates that node is a composite of multiple genes mapping to the same name in the dataset. C next to a line indicates that connection is a manually curated finding from scientific literature, while E next to a line represents a connection that was made through automated curation with secondary review by an expert.

Analysis of differentially regulated canonical pathways revealed a prominent inflammatory signature in response to bacterial treatment, with notable enrichment of pathogen-induced cytokine storm signaling (−log(*p*-value) = 11.92), z-score = +2.502). Despite lacking clear enrichment at the individual gene level, IPA also predicted activation of various inflammatory signaling, including TLR signaling (−log(*p*-value) = 6.845, activation of z-score = +3.526), NF-κB signaling (−log(*p*-value) = 14.480, activation of z-score = +5.858), and MAPK signaling (−log(*p*-value) = 8.013, activation of z-score = +2.456). Consistent with these pathway predictions, a suite of cytokines and chemokines associated with inflammatory recruitment and immune modulation were significantly upregulated. These include *TNF* (−log(*p*-value) = 2.438, log_2_FC = +3.76) and *IL1B* (−log(*p*-value) = 1.848, log_2_FC = +2.07), both implicated in acute phase inflammation and colitis-associated cancer. Chemokines such as *CCL2* (−log(*p*-value) = 1.662, log_2_FC = +3.58), *CCL5* (−log(*p*-value) = 2.364, log_2_FC = +1.98), *CCL20* (−log(*p*-value) = 1.646, log_2_FC = +2.79), *CXCL8* (IL8) (−log(*p*-value) = 6.094, log_2_FC = +4.39), *CXCL1* (−log(*p*-value) = 2.245, log_2_FC = +2.48), and *CXCL10* (−log(*p*-value) = 1.456, log_2_FC = +2.21) also had elevated expression. Functional predictions suggest that these factors contribute to the recruitment of T cells (CXCL8, CXCL10, CCL2, CCL5), monocytes (CCL2), and neutrophils (CXCL8), while IL7 enhanced Th1 cell differentiation, further shaping the immune landscape toward a pro-inflammatory state (Figure 3). Additionally, the top upstream regulator was identified as CSF2 (−log(*p*-value) = 3.041, log_2_FC = +3.92), a cytokine central to the recruitment and activation of myeloid-derived cells, including neutrophils, macrophages, and other phagocytes.

#### 3.2.2. *F. nucleatum* Encourages Immune Evasion

Immune modulation by *F. nucleatum* exposure was further evidenced by significant enrichment of the Interleukin-10 (IL-10) signaling pathway (−log(*p*-value) = 9.788, z-score = +3.464).

Several cytokines upregulated in the cross-study comparison are implicated in the induction of immunosuppressive mechanisms. Specifically, elevated expression of *CSF2*, *CCL2*, and *IL1B* was associated with the recruitment and accumulation of myeloid-derived suppressor cells (MDSCs), while CCL2 was predicted to stimulate the proliferation of tumor-associated macrophages (TAMs).

#### 3.2.3. *F. nucleatum* Influences DNA Damage and Epigenetic Modification

*F. nucleatum* exposure also triggered a significant shift in host DNA repair and chromatin regulation pathways. Most notably, IPA predicted inhibition of the core base excision repair enzyme POLB (−log(*p*-value) = 19.629, activation of z-score = −5.63) while predicted activation of components of the polycomb repressive complex 2 (PRC2), EZH2 (−log(*p*-value) = 18.606, activation of z-score = +3.46,) and SUZ12 (−log(*p*-value) = 18.932, activation of z-score = +3.35).

#### 3.2.4. *F. nucleatum* Drives Metastasis

Several pathways related to the motility and invasiveness of malignant cells were significantly enriched, including those involved in the migration of tumor cells (−log(*p*-value) = 6.777, z-score = +2.301), migration of cancer cells (−log(*p*-value) = 6.728, z-score = +1.954), movement of tumor cells (−log(*p*-value) = 6.578, z-score = +1.779), movement of cancer cells (−log(*p*-value) = 6.187, z-score = +1.105), and invasion of tumor cell lines (−log(*p*-value) = 6.025, z-score = +1.28). These pathways are fueled through cytokine and chemokine enrichment and may be coupled to the cytokine storm response (Figure 2).

This metastatic signature was accompanied by the concurrent upregulation of matrix metalloproteinases (MMPs), such as *MMP3* (−log(*p*-value) = 2.146, log_2_FC = +3.02) and *MMP13* (−log(*p*-value) = 1.103, log_2_FC = +1.37). IPA also predicted the activation of several pro-metastatic regulators, including the small GTPase RHO (−log(*p*-value) = 20.423, activation of z-score = +3.35).

### 3.3. New Discoveries

#### 3.3.1. *F. nucleatum* Influences Chemoresistance Genes and Drug Responses

Several transcriptional shifts associated with chemoresistance genes were observed following *F. nucleatum* exposure, indicating a possible interference with the efficacy of therapeutic agents, including the differential expression of *BIRC3* (Table 1).

Causal network analysis revealed a significant predicted inhibition and activation of various biological and chemical agents, including etanercept, adalimumab, infliximab, GSK2816126, and poly I:C RNA (Table 2). These compounds span anti-inflammatory biologics and epigenetic or immune-stimulating therapeutics, suggesting a broad reprogramming of host response pathways to reshape the intracellular signaling environment in a manner antagonistic to treatment efficacy.

#### 3.3.2. *F. nucleatum* Induces an Antiviral Response

**Figure 4:**
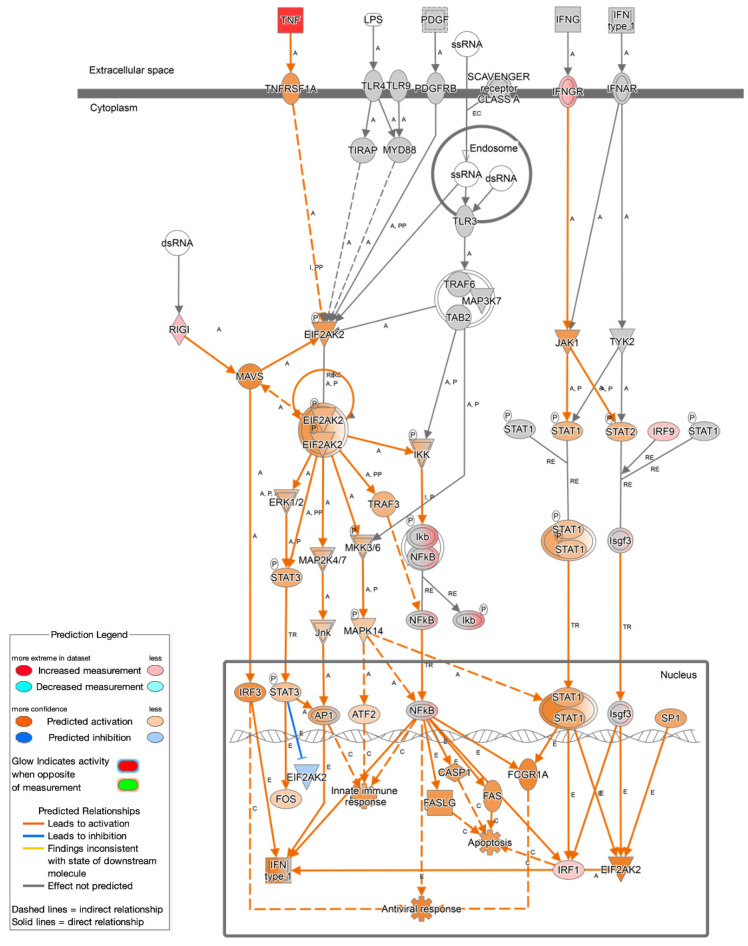
Antiviral pathways. This figure illustrates the interconnected pathways activated during the antiviral immune response. Key upstream signals such as TNF, IFN-γ, and pattern recognition receptors (e.g., IFNGR, RIG-I) initiate downstream cascades that lead to the activation of transcription factors, including NF-κB, IRF3, IRF1, and STATs. These pathways regulate the expression of genes involved in antiviral responses, apoptosis, and innate immunity. Activation of key effectors promotes antiviral defense mechanisms, such as the induction of interferons.

Several key sensors and mediators of the type I interferon pathway were predicted to be activated, including TLR3 (−log(*p*-value) = 7.695, activation of z-score = +3.284), TLR7 (−log(*p*-value) = 7.465, activation of z-score = +3.9), and IFIH1 (MDA5) (−log(*p*-value) = 19.366, activation of z-score = +6.50). Importantly, expression of *DDX58* (RIG-I), a cytosolic sensor of viral RNA, was also modestly enriched (−log(*p*-value) = 1.254, log_2_FC = +0.673), suggesting heightened surveillance through RIG-I–like receptor pathways (Figure 4).

Concurrently, type II interferon signaling was strongly activated, as evidenced by the IPA-predicted activation of IFNG (−log(*p*-value) = 20.947, activation of z-score = +5.91), along with the observed upregulation of its receptor *IFNGR* (−log(*p*-value) = 1.342, log_2_FC = +1.210) and key downstream transcription factors *IRF1* (−log(*p*-value) = 1.346, log_2_FC = +0.523) and *IRF9* (−log(*p*-value) = 2.593, log_2_FC = +0.573). While *IRF9* expression was modestly increased, IPA further predicted its functional involvement in antiviral activity and pro-inflammatory cytokine production (Figure 3).

## 4. Discussion

This study leveraged publicly available transcriptomic datasets to investigate the host response to *F. nucleatum* infection in CRC. Specifically, four independent RNAseq studies were selected based on stringent inclusion criteria. While this focused selection limits the breadth of comparative analysis, it ensures biological relevance and reflects the current availability of suitable datasets in the public domain. Although the datasets differed in cell line, MOI, and infection time, introducing variability and batch effects, this heterogeneity was strategically leveraged to identify transcriptional changes that were conserved across diverse conditions. As a result, only genes with highly significant and large differential changes in expression among all datasets were retained in the analysis. This explains why modest but biologically meaningful gene enrichments, including Wnt/β-catenin, NF-κB, and PI3K/AKT pathways that have been reported in other studies [18,45], were not identified.

Nevertheless, the integrated dataset produced here reflects core host processes consistently modulated by *F. nucleatum*, emphasizing that these mechanisms are not artifacts of a single experimental setup but likely represent fundamental features of the host–microbe interaction. While the use of in vitro cell lines does not fully represent the complexity of the tumor microenvironment, this study addresses fundamental molecular signaling pathways to inspire further in vivo explorations. This study confirms previous mechanisms for chemoresistance, being inflammation, immune evasion, DNA damage, and metastasis, as well as proposes *F. nucleatum*’s role in drug efficacy and the activation of a pseudo-antiviral state.

Our findings support and build on existing literature proposing *F. nucleatum* promotes inflammation-driven tumorigenesis, with the further implication of fostering a pro-inflammatory microenvironment that initiates chemoresistance. Previous studies report that *F. nucleatum* triggers Toll-like receptor (TLR) signaling, particularly TLR4, which in turn activates inflammatory cascades via central proteins that include NF-κB and MAPK, resulting in elevated cytokine and chemokine expression that supports immune evasion, epithelial proliferation, and carcinogenesis [13,15,46]. Although canonical components of these pathways, such as TLRs, NF-κB, or MAPKs, were not significantly differentially expressed in the integrated datasets, the downstream signaling cascades were predicted to be enriched.

Correspondingly, the expression of several downstream pro-inflammatory mediators were significantly upregulated, including *TNF*, *IL1B*, *CXCL8* (IL8), *CCL2*, *CCL5*, *CCL20*, and *CXCL10*. These cytokines and chemokines orchestrate immune cell recruitment and amplify the inflammatory response [14,47,48]. This potent recruitment signaling could establish a self-sustaining cycle of chronic inflammation that continuously attracts immune cells. Over time, instead of mounting an anti-tumor response, these cells reinforce tumor-promoting conditions. This is supported by the observation of CSF2, a cell growth-initiating cytokine, being the most significant regulatory network among these studies. CSF2 is a known biomarker and prognostic factor that influences the host immune response [49,50], and this analysis confirmed induction for the known role in recruiting and activating immune stimulatory cells. Inflammatory dysregulation of this nature not only contributes to therapeutic resistance but also facilitates tumor cell plasticity and epithelial-to-mesenchymal transition, thereby enhancing metastatic potential [14]. Collectively, these findings reinforce *F. nucleatum*’s prominent role in promoting a chronic, immune-mediated inflammatory microenvironment that may contribute to both tumor progression and resistance to therapy. Further detailed work is needed to verify this specific activity directly in vivo, perhaps with gnotobiotic animal models or organoids.

Beyond initiating inflammation, *F. nucleatum* has increasingly been recognized for its ability to manipulate the tumor immune microenvironment, and this study further supports its role in promoting immune evasion among the datasets compared. The induction of IL-10 signaling may represent a mechanism by which *F. nucleatum* dampens antitumor immune responses, thereby fostering an immunosuppressive microenvironment conducive to chemoresistance. IL-10 can inhibit pro-inflammatory cytokine production and suppress the Th1-mediated immune response, ultimately supporting tumor immune evasion. Yet, its role in cancer remains inconclusive, with studies indicating a dual role in tumor suppression and promotion. In CRC, IL-10 is significantly elevated, while in other cancers, IL-10 signaling has been shown to impair responsiveness to chemotherapy [51,52,53]. This immunomodulatory shift may enable persistent tumor cell survival during treatment, reinforcing the development of chemoresistant subpopulations and metastatic progression. The recruitment of myeloid-derived suppressor cells (MDSCs), which suppress T cell proliferation and effector function [54], has been documented as a key immunosuppressive strategy in *F. nucleatum*-associated colorectal tumors [12]. Prior studies have shown that elevated levels of IL1B, CSF2, and CCL2, all of which had increased expression in our dataset, enhance the mobilization and suppressive function of MDSCs [54,55]. CCL2 is particularly central to this process, not only recruiting MDSCs but also expanding tumor-associated macrophages (TAMs), which can shift toward an M2-like phenotype and suppress cytotoxic T cell activity [15,21,56]. Taken together, these findings underscore how *F. nucleatum* infection may foster an immunosuppressive niche by subverting both innate and adaptive immune components. By enabling immune evasion and establishing a suppressive microenvironment, *F. nucleatum* effectively shields tumor cells from immunogenic cell death, reducing the efficacy of immunotherapies and chemotherapeutic agents that rely on immune system engagement.

*F. nucleatum* has been implicated in promoting genomic instability and epigenetic modification in host epithelial cells, and this comparative analysis further suggests that this pathogen may alter host DNA repair responses. Although the genes within these pathways were not significantly enriched in this analysis, they were predicted to be activated based on downstream activity, suggesting coordinated upstream regulation and downstream induction. Prior studies have shown that *F. nucleatum* exposure increases DNA damage in colorectal epithelial cells, particularly through the generation of ROS and subsequent oxidative stress [12,13]. This damage often necessitates base excision repair (BER), a process heavily dependent on DNA polymerase β (POLB) [57]. Inhibition of *POLB*, as observed in this analysis, may impair BER efficiency and allow the accumulation of mutations, potentially driving tumorigenesis. While proposed in various studies, this work highlights the potential that a bacterium may induce mutations in mammals. Compounding this, the activation of epigenetic silencers EZH2 and SUZ12, core members of the polycomb repressive complex 2 (PRC2), suggests repression of DNA repair and tumor suppressor genes. EZH2 has been shown to transcriptionally silence key repair mediators and checkpoint regulators, contributing to genomic instability and poor prognosis in cancer [58,59]. Moreover, PRC2 activation has been linked to EMT, the acquisition of stem-like features, and silencing tumor suppressor genes that further promote cancer progression [60]. These mechanisms align with recent reports that *F. nucleatum* infection promotes a pro-carcinogenic epigenetic landscape, including altered chromatin accessibility and gene silencing at DNA repair loci [11,12]. The coordinated repression of repair enzymes and activation of epigenetic modifiers observed here highlights a dual strategy by which *F. nucleatum* may foster genomic instability, by both damaging DNA and simultaneously suppressing the cellular machinery required to correct it. These disruptions in DNA repair pathways may compromise the long-term effectiveness of DNA-damaging chemotherapies like 5-FU and oxaliplatin, common treatments for CRC [61], not by reducing their cytotoxicity, but by synergistically enhancing mutation accumulation during treatment. This may potentiate tumor evolution and relapse through this unchecked genomic instability, accelerating clonal diversification and disease progression. Considering this organism is a common inhabitant of the oral cavity and promotes mutagenic activity in the host tissue, further studies are needed that specifically examine how, where, and when this modulation occurs, as it will likely have an impact on many tissues and diseases.

As observed in analysis, *F. nucleatum* promotes a metastatic phenotype in CRC cells through the coordinated activation of cytoskeletal motility and extracellular matrix (ECM) remodeling programs. Infection is observed to induce a transcriptional state conducive to metastatic progression and EMT, with significant enrichment of pathways regulating cell migration and invasion. This metastatic gene expression profile was accompanied by the robust activation of RHO, a small GTPase that drives cytoskeletal remodeling and cell motility. This activation points to dysregulated cytoskeletal dynamics and cellular migration, processes known to be hijacked during EMT and metastatic spread [62]. Furthermore, enrichment of MMPs such as MMP3 and MMP13, which support ECM degradation and dissemination through tissue barriers, was observed in this analysis, as well as being reported in other studies [63]. MMPs also drive cancer migration through initiating EMT. MMPs have been implicated in mediating resistance to chemotherapy by altering drug penetration, modulating the tumor microenvironment, and activating pro-survival signaling pathways [63,64,65]. MMPs have been associated with *F. nucleatum* infection, facilitating microbial invasion and colonization but also contributing to tumor cell motility and immune evasion [63,65], being stimulated by cytokines such as TNF [66]. Elevated *TNF* expression in this context may further contribute to endothelial permeability and barrier dysfunction, promoting not only local invasion but also potential distant metastasis [67,68]. This metastatic reprogramming may enable tumor cells to evade drug delivery, contribute to heterogeneity in drug response, and establish resistant niches at secondary sites. 

Collectively, this analysis suggests that *F. nucleatum* may contribute to the development of chemoresistance in CRC through multifaceted reprogramming of oncogenic signaling that leads to inflammation, immune evasion, DNA damage, and metastasis. Additionally, several chemoresistance-associated genes were significantly altered in response to *F. nucleatum* exposure, suggesting that this microbe may contribute in other ways directly towards therapeutic failure in host cells. Several pro-survival and chemoresistance genes were upregulated, including *TNF* (log_2_FC = +3.76, *p* = 0.0036) [69]. *CYP3A4* (log_2_FC = +1.54, *p* = 0.0143), a key enzyme involved in xenobiotic metabolism, was also significantly induced, potentially accelerating the degradation and inactivation of chemotherapeutic agents [70]. The upregulation of *ESR1* (log_2_FC = +1.80, *p* = 0.0398), encoding estrogen receptor alpha, is notable given its known involvement in resistance to hormone-targeted therapies when mutated and in CRC is linked to poor prognosis [71,72]. *BIRC3* (log_2_FC = +2.85, *p* = 0.000692), a member of the inhibitor of apoptosis proteins (IAP) family, was significantly upregulated, confirming previous studies indicating *BIRC3* enrichment in CRC. *BIRC3* enrichment is also associated with chemoresistance to 5-FU [30]. These transcriptomic shifts suggest that *F. nucleatum* orchestrates a coordinated reprogramming of host defense, apoptosis regulation, and drug response pathways, tipping the balance toward chemoresistance. This complexity indicates that narrowly focused analyses will likely miss important regulatory triggers induced by *F. nucleatum*, indicating that a broader assessment of interconnected networks is likely needed to fully appreciate the impact of *F. nucleatum*.

Considering that therapeutic drugs have molecular targets in pathways described in this work, we examined how these signaling networks would influence drug impact. Network analysis in this study indicated that *F. nucleatum* infection modulates mechanisms that are associated with a broad panel of biologic and chemical drugs, indicating that the activity for these drugs may be affected. Etanercept, infliximab, and adalimumab are TNF-α inhibitors and were significantly inhibited among the datasets, consistent with the upregulation of TNF signaling that was observed. These drugs block TNF-mediated inflammation and are often used in immunosuppressive settings [73]. Their inhibition implies that *F. nucleatum* may amplify TNF-dependent inflammation that directly opposes and could reduce the activity for those drugs.

Multiple epigenetic and metabolic regulators were also targeted. GSK2816126, an EZH2 inhibitor [74], was inhibited, implying that *F. nucleatum* may maintain pro-tumor epigenetic states by preventing PRC2 complex inhibition. Additionally, poly dA-dT and poly rI:rC-RNA, nucleic acids that trigger innate immune stimuli through cytosolic DNA and RNA sensors such as STING1 and TLR3 [35,75], were activated, indicating an engagement of innate antiviral responses. The widespread inhibition or activation of drugs targeting inflammation, epigenetics, and metabolic pathways underscores *F. nucleatum*’s capacity to remodel the tumor environment and reinforce resistance against diverse therapeutic modalities. By influencing the activity of pathways targeted by these drugs, *F. nucleatum* may actively diminish their therapeutic efficacy, suggesting a direct microbial role in shaping host drug responsiveness and chemoresistance.

The observation that *Fusobacterium nucleatum* induces both type I and type II interferon signaling adds a novel layer to our understanding of host–microbe interactions in CRC. However, while *F. nucleatum* is traditionally studied in the context of inflammation and immune evasion, there is little research investigating whether it can also engage antiviral pathways [32,33]. The antiviral response is initiated through the interferon system, comprising secreted cytokines such as type I interferons IFN-*α* and IFN-*β* and type II interferon IFN-*γ*. These cytokines can be induced through recognition of pathogen-associated molecular patterns (PAMPs) by pattern-recognition receptors (PRRs), including TLR2, TLR3, TLR7, TLR9, and IFIH1, which then induce interferon-regulatory factors such as IRF-1, IRF-3, and IRF-9 [34]. The activation of innate RNA sensors such as TLR3, TLR7, MDA5, and RIG-I, observed in this comparative analysis, mirrors mechanisms typically reserved for viral infections and may represent an immune response that fuels chronic inflammation via the induction of interferons [34,37]. RIG-I specifically is a cytosol PRR that senses nucleic acids, and *F. nucleatum* has been identified to secrete nucleic acids to activate RIG-I and trigger a pro-inflammatory response [32,33]. Sustained activation of type I interferon responses has been shown to contribute to immune exhaustion, metastasis, and tumor-promoting inflammation in several cancers [37,40].

Moreover, *F. nucleatum* was also observed to activate type II interferon signaling, with the activation of IFNG (IFN-γ) and enrichment of its corresponding receptor IFNGR. Type II interferon signaling, while not typically antiviral, plays a crucial role in shaping immune responses that support antiviral-like states through enhanced pro-inflammatory signaling, antigen presentation, and the induction of interferon-stimulated genes via STAT1-dependent pathways [76]. IFNG is known to activate immune effector pathways, but when chronically induced, can also promote immunoregulatory programs that paradoxically support tumor survival [76].

## 5. Conclusions

This integrative transcriptomic analysis, using independent in vitro inoculation studies, identified multiple integrated signaling pathways initiated by *F. nucleatum.* These analyses uncovered observations that lead to specific molecular mechanisms to allow compelling conclusions that *F. nucleatum* plays a significant role in chemoresistance development in CRC, through mechanisms of inflammation, immune evasion, DNA damage, and metastasis. Furthermore, these findings broaden the paradigm of *F. nucleatum* pathogenicity, suggesting that its presence may alter drug efficacy and trigger a pseudo-viral immune state. The broad impact of *F. nucleatum* on the cellular response highlights the controversial role of this bacterium. Further exploration of these pathways could provide new insights into how microbial pattern recognition intersects with tumor immunology and how disrupting these signals might restore immune equilibrium and improve therapeutic outcomes.

## Figures and Tables

**Table 1:** Chemoresistance genes identified in IPA to be enriched or inhibited when co-cultured with *F. nucleatum*.

Gene	log_2_FC	Expr *p*-Value	Location	Family
*BIRC3*	2.846	6.92 × 10^−5^	Cytoplasm	enzyme
*CEBPB*	0.690	0.0309	Nucleus	transcription regulator
*CERS4*	−3.002	0.0117	Cytoplasm	transcription regulator
*CSAG2*	−4.665	0.00310	Other	other
*CYP3A4*	1.536	0.0143	Cytoplasm	enzyme
*ESR1*	1.805	0.0398	Nucleus	ligand-dependent nuclear receptor
*IL7*	1.399	0.00405	Extracellular Space	cytokine
*KISS1*	0.602	0.0667	Cytoplasm	other
*LGALS1*	−1.491	0.0502	Extracellular Space	other
*MAST1*	−1.810	0.0125	Cytoplasm	kinase
*MDK*	−1.896	0.00187	Extracellular Space	growth factor
*MIR100HG*	−2.300	0.0631	Other	other
*NFKBIA*	1.309	0.0211	Cytoplasm	transcription regulator
*PHGDH*	0.417	0.0427	Cytoplasm	enzyme
*SOD2*	0.733	0.0881	Cytoplasm	enzyme
*TMEM40*	−2.015	0.0452	Other	other
*TNF*	3.758	0.00365	Extracellular Space	cytokine
*TRIB3*	0.602	0.0824	Nucleus	kinase
*TRIM9*	−2.473	0.0154	Cytoplasm	enzyme
*VEGFA*	0.691	0.0951	Extracellular Space	growth factor

**Table 2:** Top 20 drugs recognized in causal networks to be activated or inhibited. The activation z-score predicts the likely activation or inhibition of a pathway or regulator based on the direction and consistency of gene expression changes, while the *p*-value quantifies the statistical significance of the overlap between the dataset and known pathway targets.

Compound or Drug	Molecule Type	Predicted Activation	Activation z-Score	*p*-Value
etanercept	biologic drug	Inhibited	−5.422	1.18 × 10^−31^
adalimumab	biologic drug	Inhibited	−6.075	4.12 × 10^−31^
infliximab	biologic drug	Inhibited	−6.139	5.08 × 10^−31^
tetrandrine	chemical drug	Inhibited	−6.022	7.91 × 10^−31^
poly rI:rC-RNA	biologic drug	Activated	6.401	4.1 × 10^−25^
GSK583	chemical drug	Inhibited	−4.737	2.88 × 10^−22^
ferric hexacyanoferrate(II)	chemical drug	Inhibited	−4.589	3.66 × 10^−22^
SKLB023	chemical reagent	Inhibited	−4.851	7.64 × 10^−22^
dexamethasone	chemical drug	Inhibited	−3.402	4.1 × 10^−21^
GSK2816126	chemical drug	Inhibited	−3.213	1.7 × 10^−20^
ML385	chemical reagent	Activated	3.523	1.75 × 10^−20^
L 655238	chemical reagent	Inhibited	−4.5	1.9 × 10^−20^
MDK4882	chemical reagent	Inhibited	−4.481	2.23 × 10^−20^
poly dA-dT	chemical reagent	Activated	4.781	7.24 × 10^−20^
2-(4-acetoxyphenyl)-2-chloro-N-methylethylamine	chemical reagent	Inhibited	−5.657	7.96 × 10^−20^
manumycin A	chemical reagent	Inhibited	−2.83	9.08 × 10^−20^
epicatechin	chemical drug	Inhibited	−5.584	9.19 × 10^−20^
imipramine blue	chemical drug	Inhibited	−4.737	9.54 × 10^−20^
indoxam	chemical reagent	Inhibited	−4.993	1.16 × 10^−19^
1-docosapentaenoylglycerol	chemical reagent	Inhibited	−4.258	1.21 × 10^−19^

## Data Availability

The original datasets are available at NCBI. The specific data used for this analysis are available as Supplementary Files.

## Supplementary Materials

The following supporting information can be downloaded at: https://www.mdpi.com/article/10.3390/cancers17193247/s1, File S1: A combined dataset.

## Abbreviations

The following abbreviations are used in this manuscript:

BER	Base Excision Repair
CRC	Colorectal Cancer
ECM	Extracellular Matrix
EMT	Epithelial-to-Mesenchymal Transition
GEO	Gene Expression Omnibus
IPA	Ingenuity Pathway Analysis
MDSCs	Myeloid-Derived Suppressor Cells
MMPs	Matrix Metalloproteinases
MOI	Multiplicity of Infection
PAMPs	Pathogen-Associated Molecular Patterns
PRRs	Pattern-Recognition Receptors
TAMs	Tumor-Associated Macrophages

## Appendix A

**Table A1:** Paper characteristics for the selected datasets. This table indicates their accession number on NCBI GEO, paper publication date, cancer model, cancer cell line, *Fusobacterium* strain, infection time in hours, MOI, and paper conclusions. Based on these experimental conditions, there is initial diversity between datasets as different CRC cell lines and infection times were used among the different datasets.

Accession	Pub Date	Cancer	Cell Line	Fusobacterium Strain	Infection Time (hrs)	MOI	Paper Conclusions	Citation
GSE245617	August, 2024	CRC	HCT116	*F. nucleatum* 223,726	3	100	Adhesion RadD directly binds to CD147 and induces a PI3K–AKT–NF–κB–MMP9 cascade that heightens tumorigenesis	[77]
GSE173549	October, 2021	CRC	LOVO	*F. nucleatum* 25,586	24	100	*F. nucleatum* influences the miR-1322/CCL20 axis and M2 macrophage polarization to encourage metastasis and reprogram the tumor microenvironment	[78]
GSE90944	July, 2017	CRC	HT29	*F. nucleatum* 25,586	2–4	100	*F. nucleatum* induces autophagy to encourage chemoresistance	[28]
GSE175593	October, 2021	CRC	DLD-1	*F. nucleatum* 25,586	Overnight	10	*F. nucleatum* induces *ANGPTL4* to increase glycolysis activity and thus initiate further *F. nucleatum* colonization	[79]
